# Development of a Phycocyanin–Copper-Loaded Gelatin–Chitosan Active Forming Film Solution and Its Application in Fruit Quality Preservation

**DOI:** 10.3390/foods15173039

**Published:** 2026-08-28

**Authors:** Zhicong Wang, Shanshan Ding, Yixu Wang, Wenhui Deng, Yinan Du, Wentao Su, Di Wu

**Affiliations:** 1 SKL of Marine Food Processing & Safety Control, National Engineering Research Center of Seafood, Collaborative Innovation Center of Seafood Deep Processing, Dalian Polytechnic University, Dalian 116034, China; 18237138753@163.com (Z.W.); 15045709550@163.com (S.D.); 13234215787@163.com (Y.W.); dyn7381@163.com (Y.D.); suwentao2020@yeah.net (W.S.); 2 School of Food Science and Technology, Dalian Polytechnic University, Dalian 116034, China; dengwenhui2004@163.com

**Keywords:** phycocyanin, active composite film, antioxidant, antimicrobial, grape preservation

## Abstract

Phycocyanin–Cu^2+^-loaded gelatin–chitosan composite films (Gel/Cu@PC/Ch) were developed as active packaging materials to address the postharvest deterioration of fresh produce. A series of composite films with varying chitosan (Ch) concentrations (0.1%, 0.5%, 1%, 5%, *w*/*v*) were systematically fabricated and characterized. Scanning electron microscopy (SEM) revealed that the Gel/Cu@PC/Ch_0.5_ film (0.5% chitosan) exhibited smooth, pore-free, and crack-free surface and cross-sectional morphologies, while a higher chitosan dosage (5%) triggered severe structural cracking. Gel/Cu@PC/Ch_0.5_ achieved the maximum water contact angle of 101.13 ± 9.59°, demonstrating superior surface hydrophobicity. All composite films possessed outstanding UV-shielding capacity; meanwhile, ABTS and DPPH free radical scavenging rates rose gradually with increasing chitosan content, reaching 79.71 ± 0.72% and 84.91 ± 1.31% at 5% Ch, respectively. In antibacterial assessments, Gel/Cu@PC/Ch_0.5_ showed effective inhibition against both *Staphylococcus aureus* and *Escherichia coli*, with inhibition zone diameters of 13.83 ± 0.42 mm and 14.93 ± 0.83 mm, respectively, owing to the synergistic bactericidal effect of Cu^2+^ and chitosan. In grape preservation trials conducted over 12 days, Gel/Cu@PC/Ch_0.5_ wrapped grapes exhibited the lowest weight loss (3.60 ± 0.30%), retained the highest residual firmness (316.16 ± 13.81 g) and total soluble solids (TSS), and maintained a significantly higher TAC value of 0.71 ± 0.12 nM after 12 days of storage, with no visible microbial spoilage or severe shriveling observed throughout storage. These results demonstrate that the Gel/Cu@PC/Ch_0.5_ composite film achieves a desirable integration of moisture barrier performance, antioxidant activity, and broad-spectrum antibacterial efficacy, offering a scalable and biodegradable platform for active food packaging in fresh fruit preservation.

## 1. Introduction

Fruits are rich in essential nutrients and bioactive compounds, such as vitamins, trace elements, and phenolic substances, which play crucial roles in maintaining human health and preventing chronic diseases [1]. As such, fruits have become an indispensable component of the daily human diet. However, postharvest storage of fruits is frequently accompanied by synergistic deterioration induced by moisture gradient migration, enhanced respiratory metabolism, and microbial colonization [2]. These factors collectively lead to the degradation of the mechanical strength of fruit tissue, pathogenic decay, and a subsequent decline in nutritional quality. Consequently, high postharvest loss rates of fruits are commonly observed worldwide, which not only causes substantial economic losses but also poses serious challenges to the sustainable development of the agricultural economy [3]. Therefore, extending the postharvest storage period of fruits, reducing microbial contamination, and preserving their inherent nutritional value have become urgent issues to be addressed in the field of food preservation. Traditional fruit preservation strategies primarily rely on chemical preservatives, such as sulfur dioxide and synthetic antioxidants. However, chemical agents have increasingly raised safety concerns due to potential residues and adverse effects on human health, resulting in low consumer acceptance and strict regulatory restrictions in many regions [4,5]. Against this backdrop, the development of natural, safe, and environmentally friendly novel fruit preservation technologies has emerged as an inevitable trend in the food industry, attracting extensive attention from researchers and industry professionals alike.

Food packaging films, as a physical preservation approach, exhibit remarkable advantages in terms of naturalness and safety, making them widely used in postharvest fruit preservation [6]. However, the majority of existing food packaging films are primarily fabricated from non-renewable petroleum-based synthetic polymer materials. These traditional plastic products are inherently non-biodegradable, leading to an over-reliance on end-of-life disposal methods such as incineration and landfilling [7]. Such disposal processes not only exacerbate the consumption of non-renewable energy but also contribute to a series of environmental issues, including microplastic pollution, soil acidification, and greenhouse gas emissions. Consequently, the development of environmentally friendly, bio-based, and biodegradable packaging materials to replace conventional petroleum-based plastics has become an urgent and critical challenge in the field of food packaging, driving extensive research efforts worldwide.

Bio-based biodegradable packaging materials, especially those derived from natural polysaccharides and proteins, such as gelatin, chitosan, and zein, have been recognized as the most promising alternatives to traditional petroleum-based polymers [8]. Among them, chitosan (Ch), a deacetylated derivative of chitin, exhibits excellent film-forming ability, biodegradability, biocompatibility, and broad-spectrum antimicrobial activity [9]. Ch has been widely used in the pharmaceutical and food industries and is considered an ideal substrate for bio-based food packaging [10]. Gelatin (Gel) has also been extensively studied as a material for preparing edible films owing to its good biocompatibility, degradability, and excellent film-forming properties [11]. Furthermore, the strong hydrophilicity of Gel enables efficient incorporation of water-soluble Ch, facilitating the formation of composite films with improved structural properties [12,13]. However, single-component Gel/Ch-based films still exhibit significant limitations in key functional indicators, such as antioxidant activity and antimicrobial efficacy [14,15]. These shortcomings severely restrict their practical application in food preservation, where multifunctional performance is highly demanded. Phycocyanin is a natural blue pigment with favorable antioxidant, anti-inflammatory, and immunomodulatory activities, widely reported in food and biomedical fields. Phycocyanin is also commercially produced from *Spirulina platensis* at relatively low cost, making it feasible for large-scale application in active packaging [16]. But it suffers from poor stability and easy degradation. Our previous study indicated that the introduction of Cu^2+^ into phycocyanin (PC) to form a PC-Cu^2+^ complex (Cu@PC) could significantly enhance the stability of PC and reduce its degradability [17]. Therefore, the incorporation of the phycocyanin–Cu^2+^ (Cu@PC) complex into Gel/Ch films is expected to effectively overcome the functional drawbacks of single Gel/Ch-based materials, enhance the comprehensive performance of the composite films, and thereby improve their food preservation capability. Based on this, the present study aims to develop a Cu@PC-loaded Gel/Ch active composite film and systematically evaluate its potential application in postharvest fruit preservation.

## 2. Materials and Methods

### 2.1. Materials and Chemicals

Gelatin from fish skin was purchased from Aldrich Sigma Chemical Co., Ltd. (St Louis, MO, USA). Phycocyanin (PC) from Arthrospira (Spirulina), with a purity of A_620_/A_280_ > 2.0, was obtained from Zhejiang Binmei Biotechnology Co., Ltd. (Linhai, China). Chitosan (Ch, deacetylation degree ≥95%) was purchased from Sigma-Aldrich (St. Louis, MO, USA). Cu_2_SO_4_·2H_2_O was purchased from Shanghai MACKLIN Technology Co., Ltd. (Shanghai, China). Bertani (LB) agar and LB broth were supplied by Qingdao High-tech Industrial Park Haibo Biotechnology Co., Ltd. (Qingdao, China). 2,2-Diphenyl-1-picrylhydrazyl (DPPH, CAS: 1898-66-4, purity: 96%) was obtained from Macklin Co., Ltd. (Shanghai, China). 2,2′-Azinobis-(3-ethylbenzthiazoline-6-sulphonate) (ABTS, CAS: 30931-67-0, purity: ≥98%) was supplied by Shanghai Aladdin Bio Chem Technology Co., Ltd. (Shanghai, China). Cu_2_SO_4_·2H_2_O, NaOH and HCl were purchased from Tianjin Damao Chemical Reagent Co., Ltd. (Tianjin, China). All other chemicals and reagents used in this study were of analytical grade. Total antioxidant capacity assay kits (T-AOC) (A015-2-1) were purchased from Nanjing Jiancheng Bioengineering Institute (Nanjing, China).

### 2.2. Preparation of the Gel/Cu@PC/Ch Composite

For film preparation, 15 mg of PC was dissolved in 3 mL of deionized water, followed by magnetic stirring (YUHUA, 98-2, Gongyi, China) for 2 h to ensure complete dissolution. As suggested by Li, copper ions (3.0 mM) were added to the PC solution (20 μM) to achieve a final Cu^2+^/PC molar ratio of 150:1, which was named Cu@PC nanoparticles [17]. A series of polymer solutions were prepared in 0.6 mL deionized water, each containing 90 mg gelatin, along with varying amounts of chitosan (3 mg, 15 mg, 30 mg, 150 mg) and 30 mg glycerol. Specifically, gelatin and chitosan were blended individually and dissolved in 0.6 mL of deionized water. Each mixture was stirred for 1 h, after which glycerol was incorporated. Subsequently, all mixtures were combined with 2.4 mL Cu@PC dispersion and magnetically stirred for an additional 2 h to achieve homogeneity.

The mixtures were poured onto plates and left to air-dry in a fume hood at 25 °C for 12 h. After complete drying, the films were carefully removed and stored at 25 °C under 50% relative humidity. Composite Gel/Cu@PC/Ch films were labeled according to the concentration of chitosan (Ch) as Gel/Cu@PC/Ch_0.1_, Gel/Cu@PC/Ch_0.5_, Gel/Cu@PC/Ch_1_, and Gel/Cu@PC/Ch_5_. The film without Cu^2+^ was prepared by directly mixing a 5 mg/mL PC solution with the gelatin–chitosan–glycerol mixture containing 0.5% chitosan, and was designated Gel/PC/Ch. The film without PC and Cu^2+^ was prepared by replacing the PC solution with pure water and mixing it with the same gelatin–chitosan–glycerol mixture containing 0.5% chitosan, and was designated Gel/Ch. The formulation of composite Gel/Cu@PC/Ch films is listed in Table 1.

### 2.3. Visual Appearance and Scanning Electron Microscopy (SEM) Analysis of Gel/Cu@PC/Ch Films

Macroscopic morphology of the films was captured using a Canon EOS R5 camera (Canon, Tokyo, Japan). The surface and cross-section microstructures of Gel/Cu@PC/Ch_0.1_, Gel/Cu@PC/Ch_0.5_, Gel/Cu@PC/Ch_1_, and Gel/Cu@PC/Ch_5_ films were observed using a JSM-7800F scanning electron microscope (SEM; JEOL, Tokyo, Japan) [16,17]. Samples were moved to the SEM chamber after being sputter-coated with gold. Observations were conducted at an acceleration voltage of 5.0 kV and a magnification of 2000 times.

### 2.4. Water Contact Angle Analysis of Gel/Cu@PC/Ch Films

Water contact angle (WCA) measurements were performed using a contact angle analyzer (DSA-25, Krüss, Hamburg, Germany) by depositing a 10 μL droplet of deionized water onto the film surface, and the corresponding contact angle value was recorded immediately [16].

### 2.5. UV–Visible Spectroscopic Analysis of Gel/Cu@PC/Ch Films

Ultraviolet–visible (UV–Vis) transmittance was measured using a UV–visible spectrophotometer (LAMBDA 35, PerkinElmer, Yokohama, Japan) [16]. The samples were prepared and scanned at wavelengths from 200 to 800 nm.

### 2.6. ATR-Fourier Transform Infrared Spectroscopy Analysis of Gel/Cu@PC/Ch Films

ATR-Fourier transform infrared (ATR-FTIR) spectrometry analysis was conducted at room temperature using a Thermo Scientific Nicolet iS5 (Thermo Scientific, Waltham, MA, USA) over a wavenumber range of 4000 cm^−1^ to 400 cm^−1^, with 32 cumulative scans [18,19].

### 2.7. X-Ray Photoelectron Spectroscopy Analysis of Gel/Cu@PC/Ch Films

High-resolution X-ray photoelectron spectroscopy (XPS) was performed using a Thermo Scientific Escalab 250Xi system (Thermo Scientific, Oxford, UK) with a monochromatic Al Kα X-ray source. High-resolution scans were conducted to examine the surface chemistry of the samples, as previously described [17].

### 2.8. Detection of Antioxidant Ability

The antioxidant properties of Gel/Cu@PC/Ch films were measured by DPPH and ABTS radical scavenging assays, based on previous studies [20]. In brief, the film extract was prepared by dissolving 2.5 mg of the film in 0.5 mL of deionized water. Then, 0.5 mL of 0.1 mM DPPH ethanolic solution was mixed with 0.5 mL of film extract solution and incubated in the dark for 30 min. For the ABTS assay, ABTS stock solution and working solution were prepared as described in the literature. For the ABTS assay, the ABTS stock solution and working solution were prepared according to a previous study. The working solution was reacted with Gel/Cu@PC/Ch for 3 min, and the absorbance was measured at 734 nm. Finally, the DPPH and ABTS free radical scavenging rates were calculated according to the formulation:

(1) $$Y=\frac{A\;\left(Control\right)-A\;\left(Sample\right)}{A\;\left(Control\right)}\times 100\%$$

A_(Control)_ was defined as the absorbance of DPPH/ABTS in the absence of antioxidants, whereas A_(Sample)_ was the absorbance after the addition of Gel/Cu@PC/Ch.

### 2.9. Determination of Antibacterial Activity of Gel/Cu@PC/Ch Films

The antimicrobial activities of Gel/Cu@PC/Ch_0.5_, Gel/PC/Ch_0.5_, and Gel/Ch_0.5_ were evaluated by bacterial growth curve analysis, using *Escherichia coli* (*E. coli*) (ATCC 25922) and *Staphylococcus aureus* (*S. aureus*) (ATCC 29213) as model strains. Bacterial suspensions (10^5^ CFU/mL, 100 μL) were mixed with equal volumes of composite film solution (5 mg/mL) in 96-well microplates [21,22]. Continuous absorbance at 600 nm (OD_600_) was recorded at 37 °C for 24 h using a Bioscreen C MBR system. *E. coli* and *S. aureus* in the logarithmic growth phase were diluted to 4 × 10^5^ CFU/mL using LB liquid medium. Then, 100 μL of each bacterial suspension was spread onto LB agar plates. Three Oxford cups were placed on each plate, and 100 μL of different composite film solutions were added into each cup. After incubation at 37 °C for 24 h, the inhibition zones were photographed, and their diameters were measured using a ruler.

### 2.10. Grape Preservation Experiments

Fresh Liaofeng grapes at commercial maturity, with uniform size and intact appearance, well-developed shape, glossy purple skin, fully accumulated flavor, and no mechanical damage, were purchased from Qianhe Market in Dalian City, Liaoning Province. The initial total soluble solids (TSS) content was 15.6 ± 0.62%, and the initial hardness was 367.23 ± 16.04 g. A total of 80 grapes were randomly divided into four groups at 20 °C: unwrapped control, Gel/Ch_0.5_, Gel/PC/Ch_0.5_, and Gel/Cu@PC/Ch_0.5_. Only the formulation containing 0.5% chitosan was selected for the grape study, because preliminary experiments showed that this composition exhibited the best balance of UV-blocking, antioxidant, and hydrophobicity among all tested chitosan ratios. This selection allowed us to focus on the most promising film for practical postharvest application. For the grape preservation experiment, each grape was individually placed in a plastic box. The inner side of the lid was covered with a pre-formed film, and the lid was closed so that the film came into direct contact with the grape surface, thereby achieving a wrapping effect. No additional drying step was required because the film had already been dried before use. The boxes were stored at 25 °C for 15 days. Grape weight was recorded every two days, and digital photographs were taken to record morphological changes during storage.

#### 2.10.1. Determination of Changes in Hardness

Hardness of grapes was determined according to our previous study [21,22], using a TA-XT texture analyzer (Stable Micro Systems, Godalming, UK) equipped with a cylindrical stainless steel probe (P/2, 2 mm diameter) in penetration mode. Five grapes were randomly selected from each group for texture analysis, and three measurements were performed on each grape. The test speed was set at 2 mm/s with a trigger force of 5 N.

#### 2.10.2. Determination of Total Soluble Solids Content

The total soluble solids (TSS) content was determined following a reported method [23]. Three grapes were randomly selected from each group for TSS testing, and each sample was tested 3 times. In brief, 10 g of grape tissue was homogenized with an equal mass of distilled water, and the homogenate was centrifuged at 1880× *g* for 10 min (TOMY, CAX-571, TOMY DIGITAL BIOLOGY Co., Ltd., Tokyo, Japan). The supernatant was collected and measured using a hand-held refractometer (HUT-32) at ambient temperature (23 ± 2 °C).

#### 2.10.3. Determination of Total Antioxidant Capacity

Three grapes were homogenized using a mechanical homogenizer. The homogenate was then centrifuged at 1880 *g* for 10 min, and the supernatant was collected and diluted 10-fold. All solutions were prepared strictly according to the instructions provided in the total antioxidant capacity (T-AOC) assay kit and were then subjected to the corresponding detection procedures.

### 2.11. Statistical Analysis

All experiments were performed in three independent experiments with technical replicates. The mean ± standard deviation (SD) of the three sets of data was assessed using one-way ANOVA and the post hoc Tukey’s HSD test by the SPSS software (SPSS 16.0, SPSS Inc., Chicago, IL, USA). The level of significance was set at *p <* 0.05.

## 3. Results and Discussion

### 3.1. Macroscopic and Microstructure of Gel/Cu@PC/Ch Films

**Figure 1:**
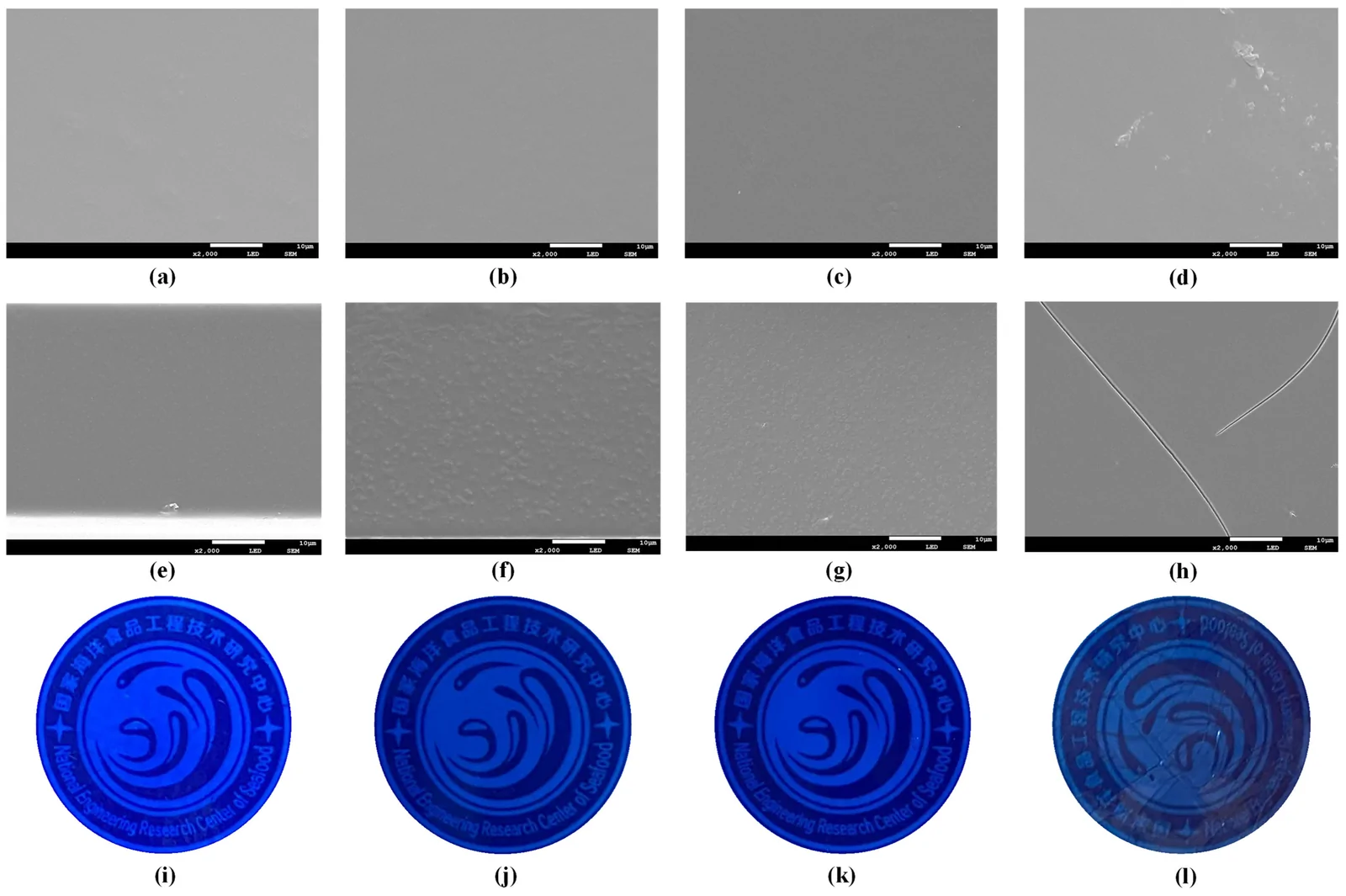
Characterization and performance of films. Surface microstructures of Gel/Cu@PC/Ch_0.1_ (**a**), Gel/Cu@PC/Ch_0.5_ (**b**), Gel/Cu@PC/Ch_1_ (**c**), and Gel/Cu@PC/Ch_5_ (**d**) films. Cross-sectional microstructures of Gel/Cu@PC/Ch_0.1_ (**e**), Gel/Cu@PC/Ch_0.5_ (**f**), Gel/Cu@PC/Ch_1_ (**g**), and Gel/Cu@PC/Ch_5_ (**h**) films (scale bar = 10 μm). Digital images showing the appearance of Gel/Cu@PC/Ch films (**i**–**l**).

The microstructures of Gel/Cu@PC/Ch_0.1_, Gel/Cu@PC/Ch_0.5_, Gel/Cu@PC/Ch_1_, and Gel/Cu@PC/Ch_5_ films were first observed by scanning electron microscopy (SEM). As shown in Figure 1i–l, the film color gradually darkened with increasing chitosan (Ch) concentration. When the Ch content reached 5%, obvious cracks appeared on the film, suggesting that the introduction of Ch affected both the color and mechanical toughness of the films. Furthermore, SEM was employed to investigate the surface and cross-sectional morphologies of the composite films, revealing the structural evolution with increasing Ch content. The surfaces of Gel/Cu@PC/Ch_0.1_ (Figure 1a) and Gel/Cu@PC/Ch_0.5_ (Figure 1b) films were smooth, compact, and continuous, with no observable cracks or pores, indicating excellent film-forming ability. Uniformly dispersed aggregates were observed on the film surface, which could be attributed to the stable complexation between PC and Cu^2+^ as reported in previous studies [17]. With further increasing chitosan proportion, the surface roughness of Gel/Cu@PC/Ch_1_ (Figure 1c) increased noticeably. When the Ch concentration was raised to 5%, distinct cracks emerged on both the surface (Figure 1d) and cross-section (Figure 1h), severely damaging the structural integrity of the film. These results further verified that excessive chitosan loading significantly alters the morphological and structural properties of the composite films.

### 3.2. Basic Properties of the Multifunctional Gel/Cu@PC/Ch Films

**Figure 2:**
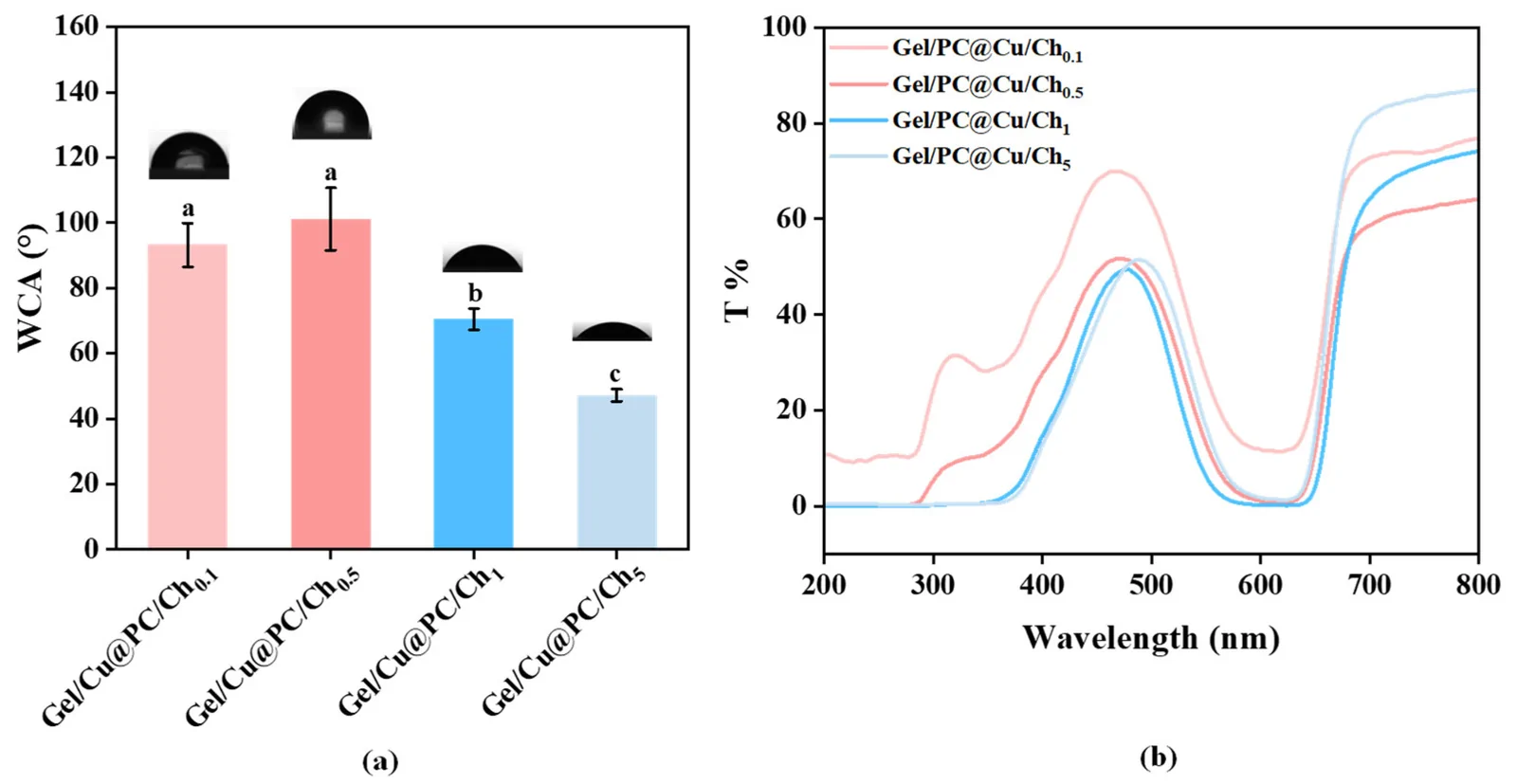
Basic properties of the multifunctional Gel/Cu@PC/Ch films. (**a**) Water contact angle (WCA) measurements, and (**b**) UV–Vis transmittance spectra of the films. Different letters in (**a**) indicated significant differences (*p* < 0.05).

Water contact angles (WCAs) of the Gel/Cu@PC/Ch films with different chitosan (Ch) contents were presented in Figure 2a. WCAs were measured to evaluate the surface hydrophilicity or hydrophobicity of the films. The Gel/Cu@PC/Ch_1_ and Gel/Cu@PC/Ch_5_ films exhibited relatively low WCAs of 70.50 ± 3.26° and 47.17 ± 1.88°, respectively, indicating hydrophilic surfaces. Notably, the WCA significantly increased with decreasing Ch content, suggesting enhanced surface hydrophobicity. The film Gel/Cu@PC/Ch_0.5_ achieved the highest WCA of 101.13 ± 9.59°, demonstrating a typical hydrophobic surface. As shown in Figure S1a, ATR-FTIR results revealed that the Gel/Cu@PC/Ch_0.5_ film possessed high infrared transmittance. As shown in Figure 2b, UV–Vis transmittance revealed that the Gel/Cu@PC/Ch composite films exhibited efficient ultraviolet (UV) blocking performance, suggesting great potential for UV-protective food packaging applications [24,25]. Compared with Gel/Cu@PC/Ch_0.1_, the Gel/Cu@PC/Ch_0.5_ film displayed superior UV-shielding capability, as reflected by its lower UV transmittance. In food packaging systems, UV resistance is essential for maintaining food quality and prolonging shelf life, since UV irradiation can trigger photodegradation of nutrients and deterioration of sensory properties [26]. The UV-blocking characteristics of these composite films are therefore considered beneficial for reducing such photooxidative damage and helping to protect packaged foods from UV exposure.

### 3.3. The Anti-Oxidation Properties of Gel/Cu@PC/Ch Films

**Figure 3:**
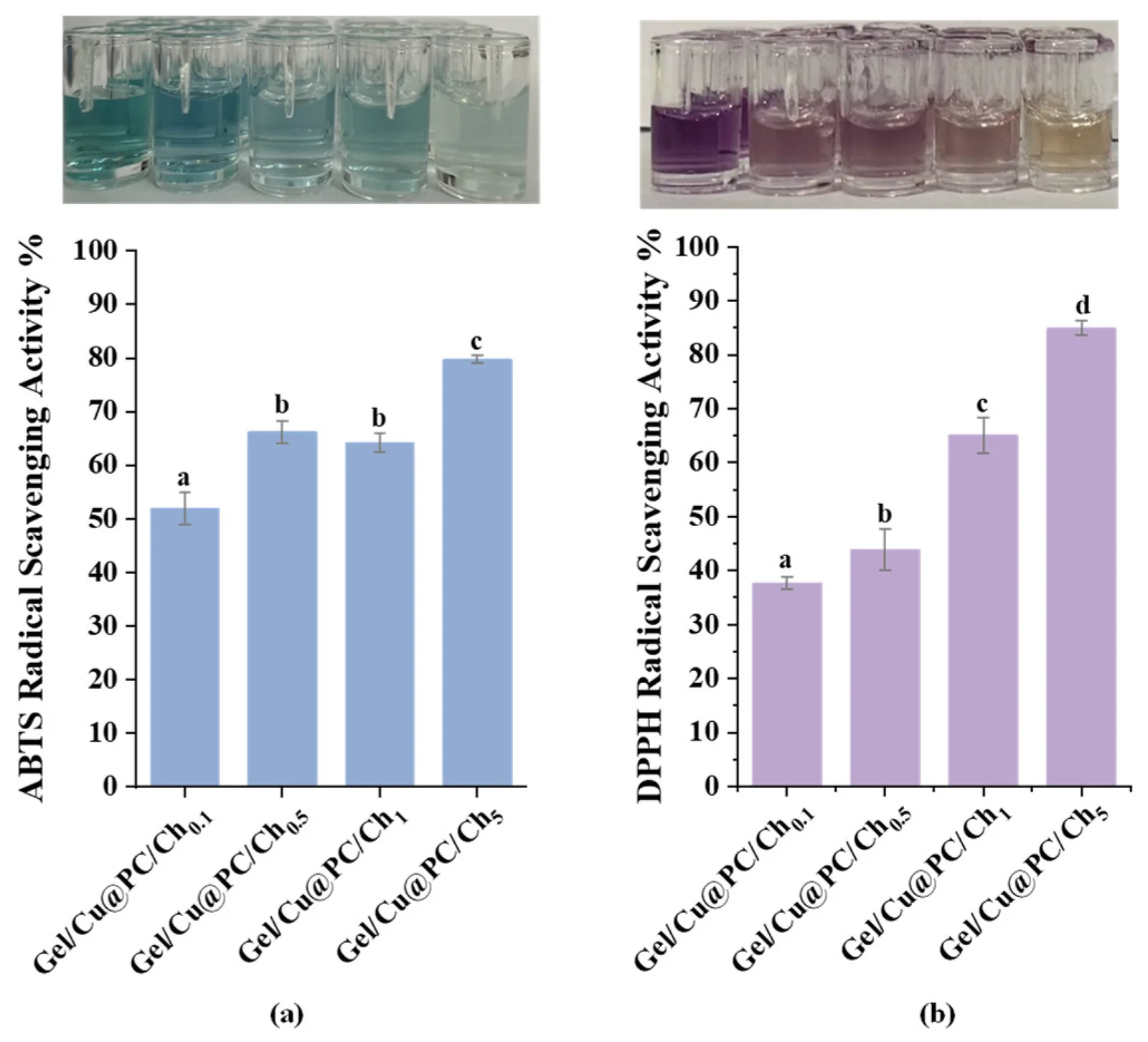
Determination of antioxidant activity. (**a**) ABTS radical scavenging activity and (**b**) DPPH radical scavenging activity of the films. Different letters indicated significant differences (*p* < 0.05).

The biological and functional properties of the Gel/Cu@PC/Ch films were mainly ascribed to their favorable antioxidant activity. As presented in Figure 3a, ABTS radical scavenging activity gradually increased with elevated Ch content. The scavenging ratios at Ch concentrations of 0.1%, 0.5%, 1%, and 5% were 51.93 ± 3.05%, 66.23 ± 2.13%, 64.26 ± 1.72%, and 79.71 ± 0.72%, respectively. A similar upward trend was observed in the DPPH radical scavenging assay, with corresponding values of 37.74 ± 1.17%, 43.89 ± 3.78%, 65.06 ± 3.20%, and 84.91 ± 1.31% (Figure 3b). Overall, measurable free radical scavenging activity was demonstrated by all formulations, with the most pronounced effect being observed at 5% Ch content [27]. Nevertheless, considering the comprehensive film performance from preceding experiments, Gel/Cu@PC/Ch_0.5_ was determined to be the optimal formulation and was thus selected for subsequent mechanism exploration and practical preservation applications.

### 3.4. The Antibacterial Activity of Gel/Cu@PC/Ch Films

**Figure 4:**
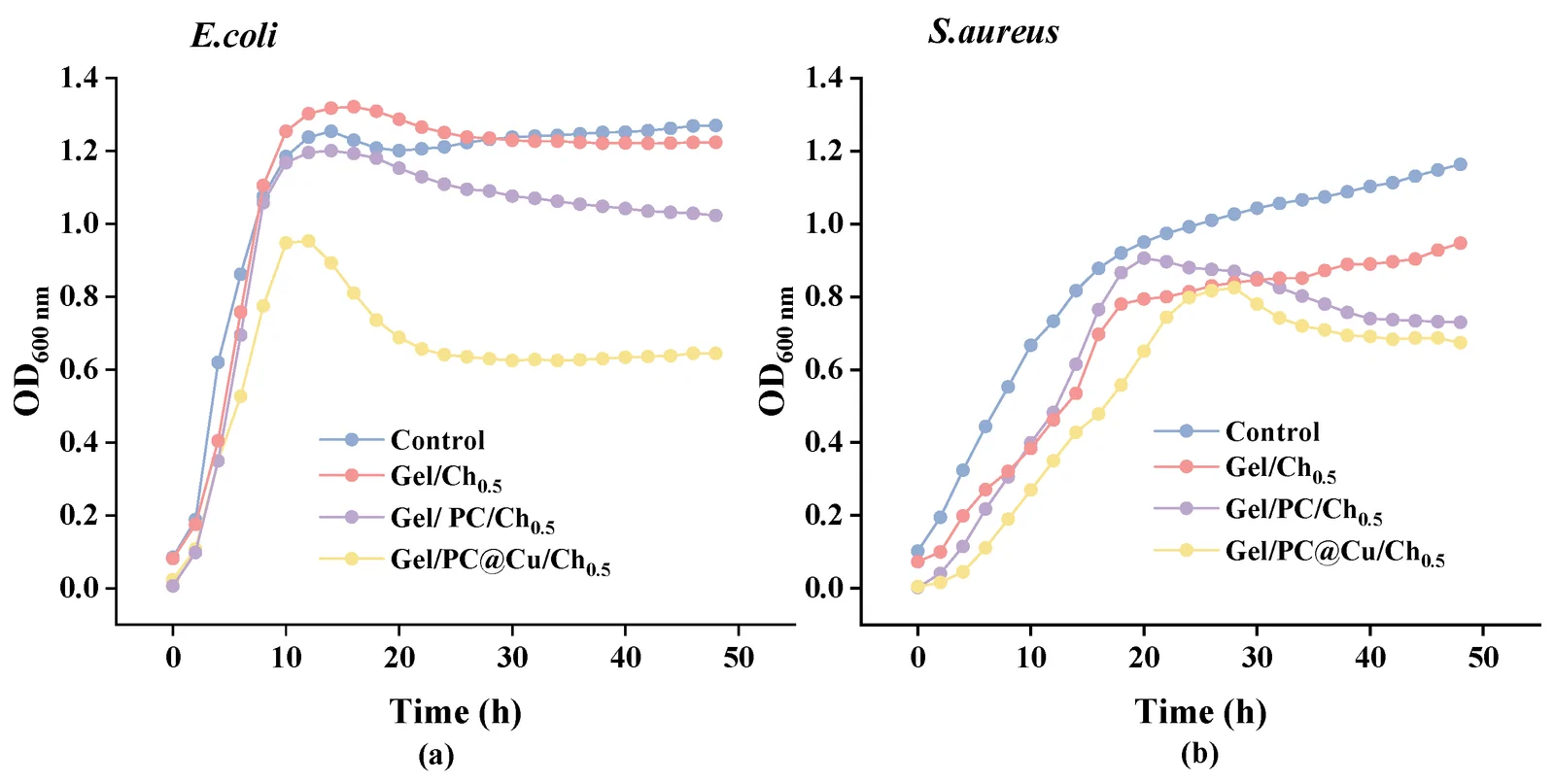
Growth curves of *E. coli* and *S. aureus* following treatment with Gel/Cu@PC/Ch films, respectively. (**a**) Growth curves of *E. coli* following Gel/Cu@PC/Ch film treatment. (**b**) Growth curves of *S. aureus* following Gel/Cu@PC/Ch film treatment.

To further evaluate the antibacterial efficacy of the composite films, bacterial growth curve analysis and inhibition zone assays were conducted on *E. coli* and *S. aureus* treated with Gel/Cu@PC/Ch_0.5_, Gel/PC/Ch, and Gel/Ch (Figure 4). As illustrated by the growth curve results, after co-incubation with *E. coli* and *S. aureus*, both Gel/Cu@PC/Ch_0.5_ and Gel/PC/Ch exhibited inhibitory effects on *S. aureus* growth, with Gel/Cu@PC/Ch_0.5_ showing a significantly stronger inhibitory capacity. In contrast, Gel/Ch displayed almost no suppressive effect on *S. aureus* proliferation [28]. Notably, only Gel/Cu@PC/Ch_0.5_ effectively inhibited *E. coli* growth, while Gel/PC/Ch and Gel/Ch failed to exert any significant inhibitory activity against *E. coli*. These results indicate that Gel/Cu@PC/Ch_0.5_ possesses potent antibacterial activity against both *E. coli* and *S. aureus*, and this activity is primarily attributed to the presence of Cu^2+^.

**Figure 5:**
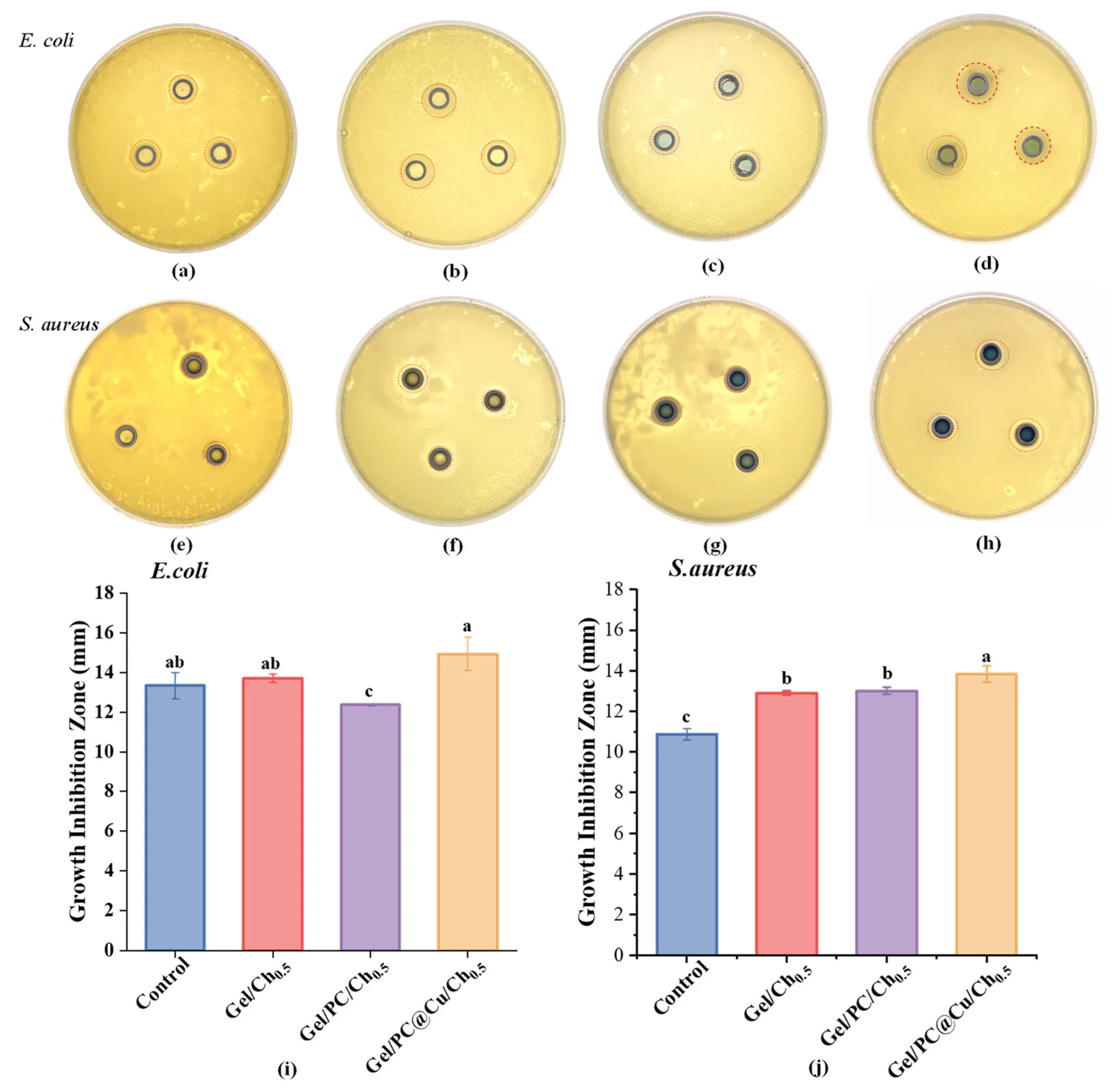
Inhibition zones of *E. coli* and *S. aureus* under different treatments. Inhibition zones of *E. coli* under different treatments. (**a**) Control, (**b**) Gel/Ch_0.5_, (**c**) Gel/PC/Ch_0.5_, (**d**) Gel/PC@Cu/Ch_0.5_. Inhibition zones of *S. aureus* under different treatments. (**e**) Control, (**f**) Gel/Ch_0.5_, (**g**) Gel/PC/Ch_0.5_, (**h**) Gel/PC@Cu/Ch_0.5_, growth inhibition zone diameter of different films (**i**,**j**). The inhibition zones were outlined with red circles for clarity. Different letters indicated significant differences (*p* < 0.05).

For *S. aureus* (Figure 5h,j), the Gel/Cu@PC/Ch_0.5_ film exhibited an inhibition zone diameter of 13.83 ± 0.42 mm, which was significantly larger than those of the control group, the Gel/PC/Ch group, and the Gel/Ch group [29]. For *E. coli* (Figure 5d,i), the inhibition zone of the Gel/Cu@PC/Ch_0.5_ film reached 14.93 ± 0.83 mm. Collectively, these results clearly demonstrate the significant antibacterial activity of the Gel/Cu@PC/Ch_0.5_ composite film, further confirming that the introduction of Cu^2+^ is crucial for its antibacterial efficacy [30,31].

### 3.5. Application of Gel/Cu@PC/Ch Films in Preserving Grapes

#### 3.5.1. The Change in Appearance

**Figure 6:**
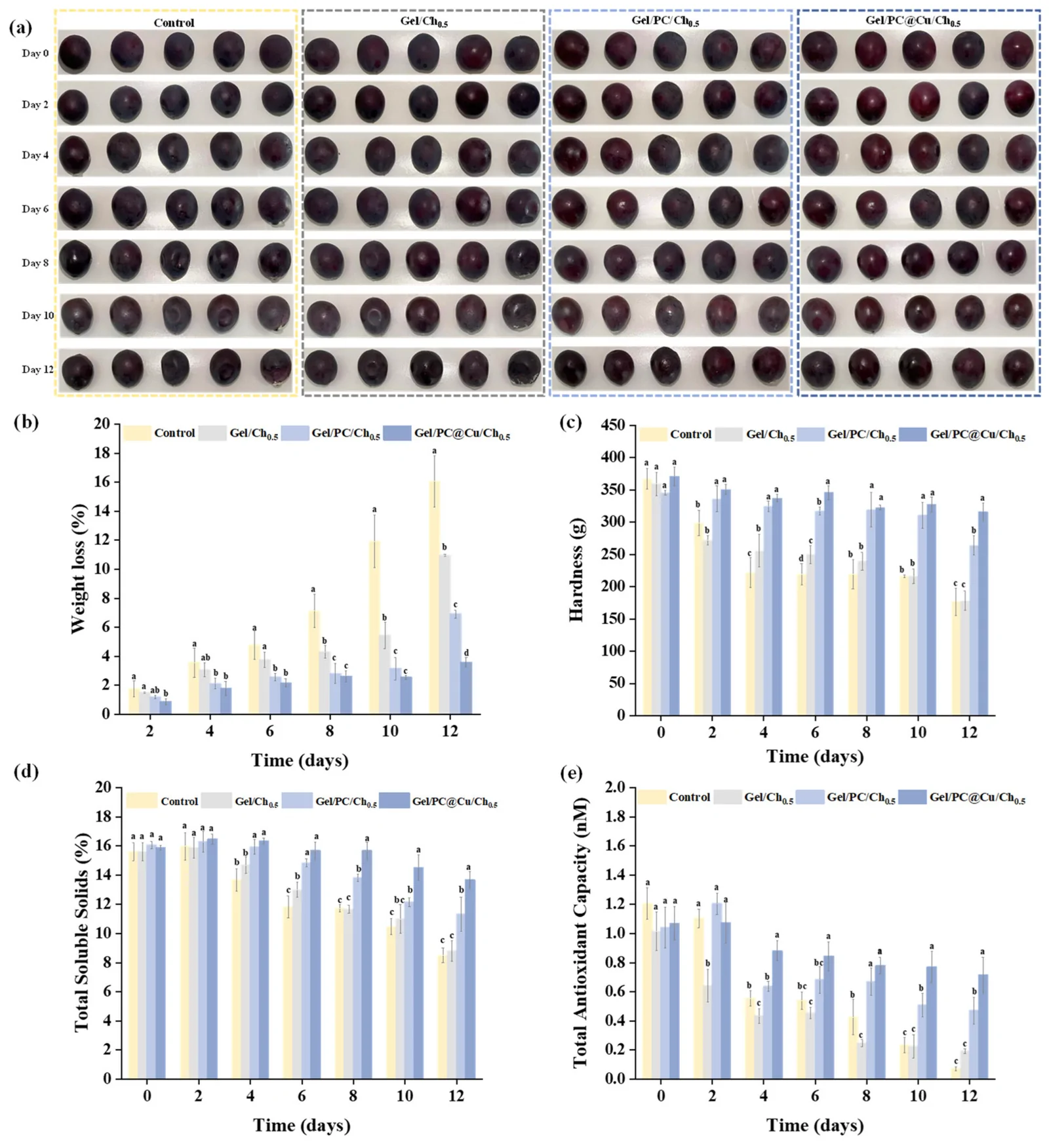
Sustained preservation of grapes treated with different films for 0, 2, 4, 6, 8, 10, and 12 days. (**a**) Appearance, (**b**) weight loss, (**c**) hardness, (**d**) total soluble solids, and (**e**) total antioxidant capacity of grapes. Different letters indicated significant differences (*p* < 0.05).

The appearance of grapes treated with different wrappings during 12 days of storage was shown in Figure 6a. By the eighth day of storage, obvious dehydration and softening were observed in the control group. In contrast, grapes with Gel/Cu@PC/Ch_0.5_ or Gel/PC/Ch films maintained a relatively intact appearance with no significant changes, indicating that the composite wrappings effectively reduced moisture loss of the grapes. Microbial growth with shriveling and discoloration was detected on the surface of control grapes on the 10th day, followed by further deterioration. Notably, grapes treated with Gel/Cu@PC/Ch_0.5_ showed minimal shrinkage and no visible microbial spoilage throughout the storage period. The excellent preservation effect of Gel/Cu@PC/Ch_0.5_ is presumably attributed to its lower oxygen permeability, which can reduce internal oxygen levels and inhibit microbial proliferation, combined with its inherent antibacterial activity [32,33]. Collectively, these results demonstrate that the Gel/Cu@PC/Ch_0.5_ effectively mitigates moisture loss and preserves the morphological integrity of grapes during storage.

#### 3.5.2. Weight Loss

Weight loss, a critical indicator of fruit freshness during storage that is mainly associated with water evaporation and respiratory metabolism, gradually increased over the 12-day storage period across all treatment groups (Figure 6b) [34]. By the 12th day of storage, the weight loss rate of the control group reached 16.07 ± 1.76%, which was significantly higher than those of all film groups. Grapes wrapped with Gel/Ch exhibited better water retention capacity compared to the control group but still failed to effectively suppress weight reduction. Notably, the Gel/Cu@PC/Ch_0.5_ and Gel/PC/Ch groups showed significantly lower weight loss rates relative to the control group, indicating that the incorporation of PC effectively enhanced the moisture barrier properties of the composite films and slowed down water loss. Furthermore, the Gel/Cu@PC/Ch_0.5_ group exhibited the lowest weight loss rate (3.60 ± 0.30%) on day 12, which was significantly lower than that of the Gel/PC/Ch group. This finding suggests that the introduction of Cu^2+^ further improved the moisture barrier performance of the composite film. The superior preservation effect of Gel/Cu@PC/Ch_0.5_ is presumably attributed to its lower oxygen permeability, which can reduce internal oxygen levels and inhibit microbial proliferation, coupled with its inherent antibacterial activity. Collectively, these results confirm that the Gel/Cu@PC/Ch_0.5_ wrapping effectively mitigates moisture loss and maintains the postharvest quality of grapes during storage.

#### 3.5.3. Hardness

Hardness, another critical quality parameter reflecting fruit ripening and structural integrity, was monitored throughout the 12-day storage period. As shown in Figure 6c, the control group exhibited a significant decrease in hardness, declining from an initial value of 367.23 ± 16.04 g (day 0) to 176.47 ± 21.43 g (day 12). In contrast, grapes with the Gel/Cu@PC/Ch_0.5_ film maintained significantly higher hardness during storage, retaining a value of 316.16 ± 13.81 g on day 12. This notable difference indicates that the Cu@PC complex within the composite film, which acts as an effective water vapor barrier, likely played a pivotal role in preserving the structural integrity of grape tissue [35]. By reducing moisture loss and inhibiting excessive softening, the Gel/Cu@PC/Ch_0.5_ wrapping effectively delayed the postharvest senescence of grapes, consistent with the previously observed superior moisture retention and preservation effects.

#### 3.5.4. Total Soluble Solids

To further explore the internal quality variations in grapes during storage, changes in total soluble solids (TSS) content were monitored, as shown in Figure 6d. TSS is a vital nutritional index for evaluating fruit quality, and its variation is closely related to respiratory metabolism and microbial growth [36]. The TSS content initially increased, which was attributed to the continued maturation of grapes at the early storage stage, followed by a gradual decline thereafter [37]. Throughout the entire storage period, the Gel/Cu@PC/Ch_0.5_ group maintained a consistently higher TSS level than all other treatment groups. This result indicates that the Gel/Cu@PC/Ch_0.5_ wrapping could effectively inhibit fruit respiration and microbial contamination, thereby slowing the consumption of nutrients and better preserving the internal quality of grapes.

#### 3.5.5. Total Antioxidant Capacity

**Figure 7:**
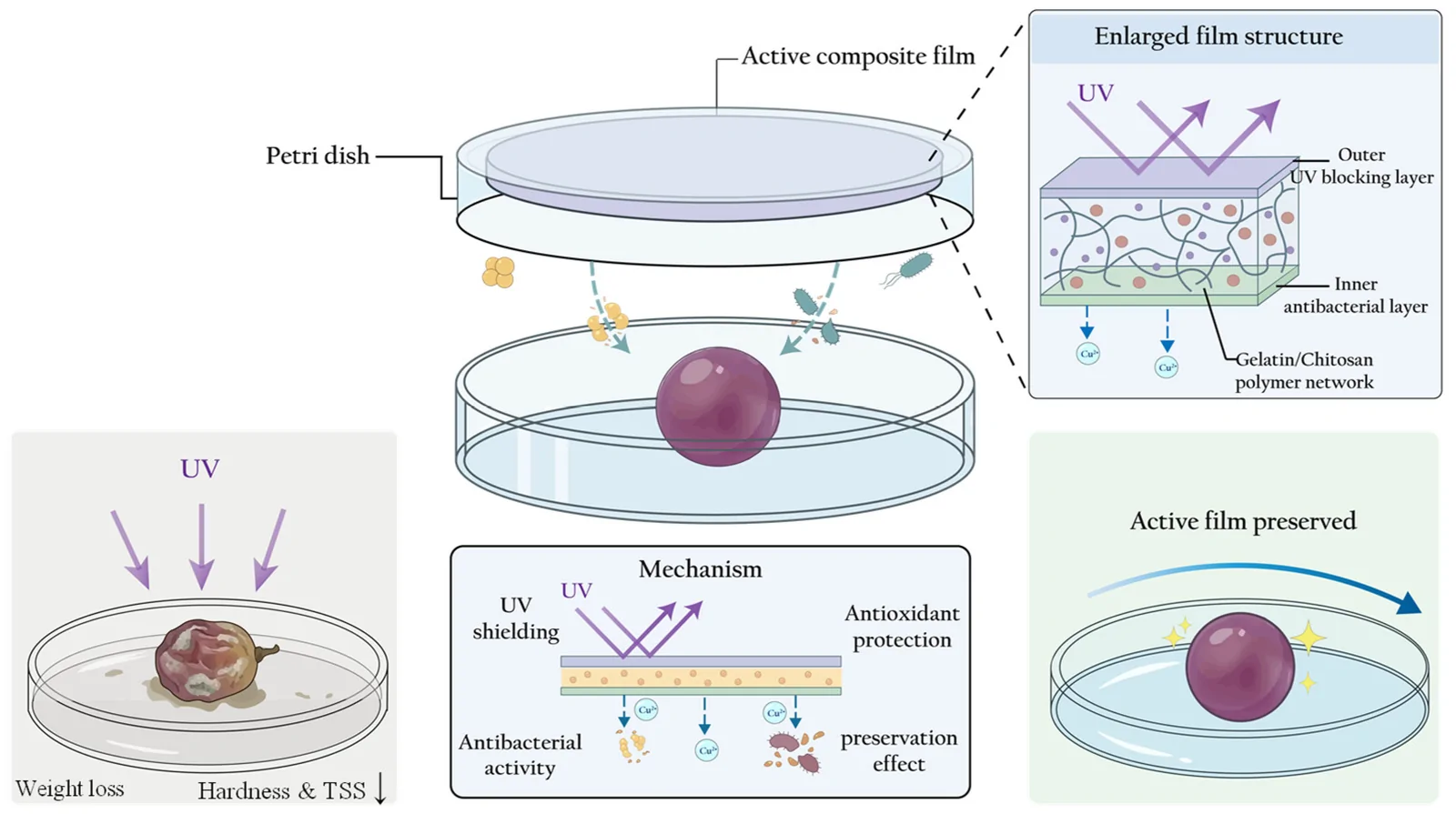
Preparation and multifunctional mechanisms of the film.

Total antioxidant capacity (TAC), a key indicator reflecting the nutritional quality and oxidative stability of grapes, was determined throughout the 12-day storage period. As shown in Figure 6e, the initial TAC of the control group was 1.20 ± 0.11 nM. After 12 days of storage, the TAC of the control group decreased significantly to 0.07 ± 0.01 nM, indicating severe oxidative degradation of antioxidant components during storage [38]. In contrast, the Gel/Ch and Gel/PC/Ch wrapped groups retained slightly higher TAC values, with 0.19 ± 0.02 nM and 0.47 ± 0.09 nM recorded on day 12, respectively. Notably, grapes wrapped with the Gel/Cu@PC/Ch_0.5_ film maintained a significantly higher TAC of 0.71 ± 0.12 nM at the end of storage, which was substantially higher than those of the other wrapped groups. This finding suggests that the Gel/Cu@PC/Ch_0.5_ film effectively alleviates the oxidative degradation of antioxidant substances in grapes, which is presumably associated with its inherent antioxidant activity and ability to inhibit microbial growth and respiratory metabolism [39]. Since phycocyanin can effectively block UV light, it can significantly mitigate weight loss, firmness changes, variations in soluble solids content, and loss of antioxidant capacity in grapes during postharvest storage [40]. Collectively, these results confirm that the film Gel/Cu@PC/Ch_0.5_ possesses strong UV-blocking ability combined with prominent antioxidant and antibacterial activities (Figure 7), which may help retain the nutritional value and oxidative stability of grapes during postharvest storage.

## 4. Conclusions

In this study, a multifunctional active composite film was successfully fabricated based on gelatin–chitosan (Gel/Ch) incorporated with a phycocyanin–Cu^2+^ complex (Cu@PC). By systematically regulating the chitosan (Ch) content, the microstructures of the composite films were optimized, and the Gel/Cu@PC/Ch_0.5_ formulation was identified as the optimal candidate, featuring a smooth, continuous, and defect-free surface morphology that ensures excellent structural integrity. The Gel/Cu@PC/Ch_0.5_ film exhibited superior comprehensive performance, including enhanced hydrophobicity, efficient ultraviolet (UV) blocking capability, and remarkable antioxidant and antibacterial activities. In practical postharvest preservation of grapes, the Gel/Cu@PC/Ch_0.5_ film effectively delayed fruit quality deterioration by synergizing its excellent water vapor and oxygen barrier properties with inherent antioxidant and antimicrobial functions. Specifically, compared with other treatment groups, the Gel/Cu@PC/Ch_0.5_ wrapping significantly reduced grape weight loss and better maintained fruit hardness, total soluble solids (TSS) content, and total antioxidant capacity (TAC) throughout the 12-day storage period. In conclusion, this work verifies that the Gel/Cu@PC/Ch_0.5_ composite film possesses favorable antioxidant, antibacterial, and grape preservation performance, showing promising preliminary application potential for postharvest fruit fresh-keeping. It not only provides a novel and efficient strategy for developing environmentally friendly food preservation technologies but also offers a promising approach for the high-value utilization of gelatin, chitosan, and phycocyanin in the field of active food packaging.

## Figures and Tables

**Table 1:** The formulation of composite Gel/Cu@PC/Ch films.

Film Label	Gelatin (%)	Chitosan (%)	Glycerol (%)	Phycocyanin (%)	Cu (%)
Gel/Ch_0.5_	3	0.5	1	0	0
Gel/PC/Ch_0.5_	3	0.5	1	0.5	0
Gel/Cu@PC/Ch_0.1_	3	0.1	1	0.5	0.003
Gel/Cu@PC/Ch_0.5_	3	0.5	1	0.5	0.003
Gel/Cu@PC/Ch_1_	3	1	1	0.5	0.003
Gel/Cu@PC/Ch_5_	3	5	1	0.5	0.003

## Data Availability

The original contributions presented in this study are included in the article/Supplementary Materials. Further inquiries can be directed to the corresponding author.

## Supplementary Materials

The following supporting information can be downloaded at: https://www.mdpi.com/article/10.3390/foods15173039/s1. Figure S1: ATR-FTIR and high-resolution XPS spectra of the films. (a) ATR-FTIR, (b) N 1s spectrum, (c) O1s spectrum, and (d) Cu 2p spectrum.

## Abbreviations

The following abbreviations are used in this manuscript.

PC	Phycocyanin
Gel	Gelatin
Ch	Chitosan
SEM	Scanning electron microscopy
WCA	Water contact angle
TSS	Total soluble solids
TAC	Total antioxidant capacity
UV	Ultraviolet visible
ATR-FTIR	ATR-Fourier transform infrared
XPS	X-ray photoelectron spectroscopy
LB	Bertani
DPPH	2,2-Diphenyl-1-picrylhydrazyl
ABTS	2,2′-Azinobis-(3-ethylbenzthiazoline-6-sulphonate)
T-AOC	Total antioxidant capacity assay kits
CFU	Colony-forming unit
*S. aureus*	*Staphylococcus aureus*
*E. coli*	*Escherichia coli*
